# Protective effects of melatonin against nicotine-induced disorder of mouse early folliculogenesis

**DOI:** 10.18632/aging.101405

**Published:** 2018-03-28

**Authors:** Yu-Feng Wang, Xiao-Feng Sun, Ze-Li Han, Lan Li, Wei Ge, Yong Zhao, Massimo De Felici, Wei Shen, Shun-Feng Cheng

**Affiliations:** 1 College of Animal Science and Technology, Qingdao Agricultural University, Qingdao 266109, China; 2 College of Life Sciences, Institute of Reproductive Sciences, Qingdao Agricultural University, Qingdao 266109, China; 3 The First Affiliated Hospital of Chinese PLA General Hospital, Beijing 100039, China; 4 Department of Biomedicine and Prevention, University of Rome Tor Vergata, Rome 00133, Italy

**Keywords:** nicotine, melatonin, primordial follicle, ovarian reserve, ROS, autophagy

## Abstract

In this paper, we show that neonatal mice injected for five consecutive days with nicotine display impaired germ cell cyst breakdown and primordial follicle assembly resulting in decreased ovarian reserve lasting until sex maturation age. The effects of nicotine on the pups ovaries were associated with decreased expression of oocyte specific genes such as *Nobox*, *Lhx8*, *Figlα* and *Sohlh2*. Moreover, the ovaries of pups injected with nicotine showed increased level of cell oxidative stress and autophagic markers (upregulation of AMPKα-1, increased ratio LC3-II/LC3-I, downregulation of AKT and mTOR). Noteworthy, all these effects were counteracted by the administration of the hormone melatonin in 1 μM. *In vitro* culture of 0 dpp ovaries for 5 days in the presence of 10 μM nicotine reproduced its effect on germ cell cyst breakdown and primordial follicle assembly, furthermore it also revealing about 20% reduction of somatic cells proliferation, and these effects was prevented when melatonin was added to the medium. Taken together these results show that nicotine exposure can adversely affect the establishment of the ovarian reserve in the mouse likely by locally inducing cellular stress altering the primordial follicle assembly and that melatonin, however, is able to counteract such effects.

## Introduction

Smoking is a well-established risk factor for many human health conditions, including increased risk of subfertility, infertility and pregnancy loss [1,2]. The incidence of smoking has generally fallen over the last 20 years but significant numbers of women continue to smoke before and during pregnancy. Total 13-25% of pregnancies are exposed tobacco smoke in many countries [3]. There are evidences that in utero exposure to cigarette smoke has long-term consequences on the fertility of the offspring, including reproductive abnormalities and reduced fertility [2]. Indeed, the daughters of mothers who smoked during pregnancy have an earlier menopause, shorter reproductive lifespan, and reduced fecund ability [2,4,5].

Establishment of the primordial follicle (PF) pool is critical for female fertility because it represents the total population of oocytes available throughout the reproductive lifetime, and any abnormality in such process can result in infertility [6]. PF formation is a tightly regulated process that takes place in the second trimester of human gestation or shortly after birth in mouse [6,7]. After migration to the gonadal ridges at 10.5 days post coitum (dpc), female mouse primordial germ cells (PGCs), the precursors of oogonia/oocytes, divide by mitosis with incomplete cytokinesis to form germ cell cysts [2,8]. At approximately 13.5 dpc, female germ cells enter meiosis becoming primary oocytes that arrest at the diplotene stage of meiotic prophase I around birth. The majority of germ cell cysts separate into individual oocytes (cyst breakdown) after birth and surrounded by a single layer of flattened pre granulosa cells to form the PF pool [9].

Germ cell cyst breakdown and PF formation is susceptible to many compounds [10–15]. Some *in vivo* studies have demonstrated decreased PF counts coupled with oxidative stress and cell autophagy in mice exposed to cigarette smoke at concentrations comparable to those of smoker women [16–19]. Among the smoke products, which is a complex mixture of over 4000 different chemicals, nicotine is considered a major inducer of cellular oxidative stress that can perturb embryonic development [20,21]. In fact, nicotine can quickly cross the placenta to reach the embryo and accumulates in fetal blood and amniotic fluid [22]. Previous studies have shown that in utero nicotine exposure causes impaired fertility, altered ovarian steroid hormone and protein levels, and increased the numbers of atretic follicles in adult female rat offspring [23,24]. These characteristics overtly resemble the clinical profile of polycystic ovarian syndrome (PCOS), suggesting that fetal and/or neonatal exposure to nicotine may contribute to ovarian dysfunction in adult life [23]. Although there is overwhelming epidemiological evidence linking female cigarette smoke and reduction of the ovarian reserve there is no direct evidence that nicotine can impair PF assembly, the major process accountable for establishing the reserve.

Melatonin (5-methoxy-N-acetyltryptamine), as a neurohormone and highly conserved molecule, is produced in all vertebrate species. Melatonin is primarily released by the pineal gland and a powerful free radical scavenger and antioxidant [25]. It facilitates various physiological functions such as circadian rhythm functions, sexual activity and reproductive functions, tumor growth, immune response and aging [26,27]. It is interesting to note that melatonin has been found in the human follicle fluid and can be produced in the ovary and in particular by granulosa cells [28,29]. Moreover, the melatonin receptor type 1 (MT1) has been found both in oocytes and granulosa cells of various mammalian species and the hormone was reported to exert beneficial effects on the ovary under a variety of conditions [30–44].

Using *in vivo* and *in vitro* experimental approaches and the mouse model, the purpose of present study was to investigate whether nicotine affect processes crucial for the formation of the PF pool at birth and if melatonin was eventually able to counteract such effect.

## RESULTS

### Nicotine impairs folliculogenesis in pup’s ovaries

**Figure 1:**
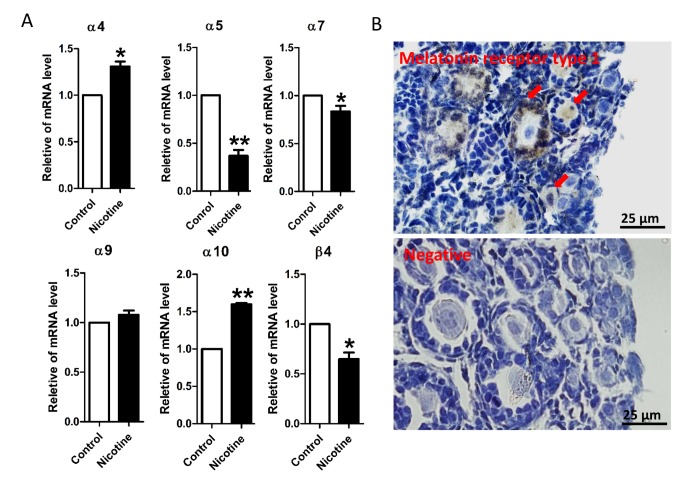
**qRT-PCR analysis of nicotinic acetylcholine receptor (nAChRs) subunit mRNA and immunolocalization of MT1 protein in the neonatal mouse ovaries.** (**A**) Relative changed of nAChR in mRNA expression in the normal control, intraperitoneally injected 1 mg/kg nicotine treated mouse ovaries. The results are presented as mean±SD. All the experiments were repeated at least three times. * P < 0.05; ** P < 0.01. (**B**) Immunolocalization of MT1 receptor in neonatal mouse ovarian follicles (arrows) and negative control.

In order to investigate whether nicotine exposure can affect early folliculogenesis in the mouse experimental model, we first analyzed the expression of nicotinic acetylcholine receptors (nAChRs) in the mouse pup’s ovaries by qRT-PCR. Of the 16 nAChRs subunits [45], six namely α4, α5, α7, α9, α10 and β4, resulted present at detectable level in the ovary. Noteworthy, after 5 days of consecutive injection of 1 mg/kg body weight (bw) nicotine (from 0 to 4 dpp), an increase in α4 and α10 at mRNA levels, and a decrease in α5, α7, and β4 mRNA in the ovaries of the treated pups were observed (Fig. 1A). Next we investigated whether such nicotine injection protocol affected the PF assembly mostly occurring in the mouse ovaries between 0-4 dpp [46,47]. None of the pups died during the nicotine injection period, while their body weights increased normally without apparent differences between the experimental groups (Figs. S1A, S1B). We observed, however, that the ovaries of mice injected with the higher dose of nicotine appeared smaller in size in comparison to control and low nicotine dose treated pups (Fig. S1C).

**Figure 2:**
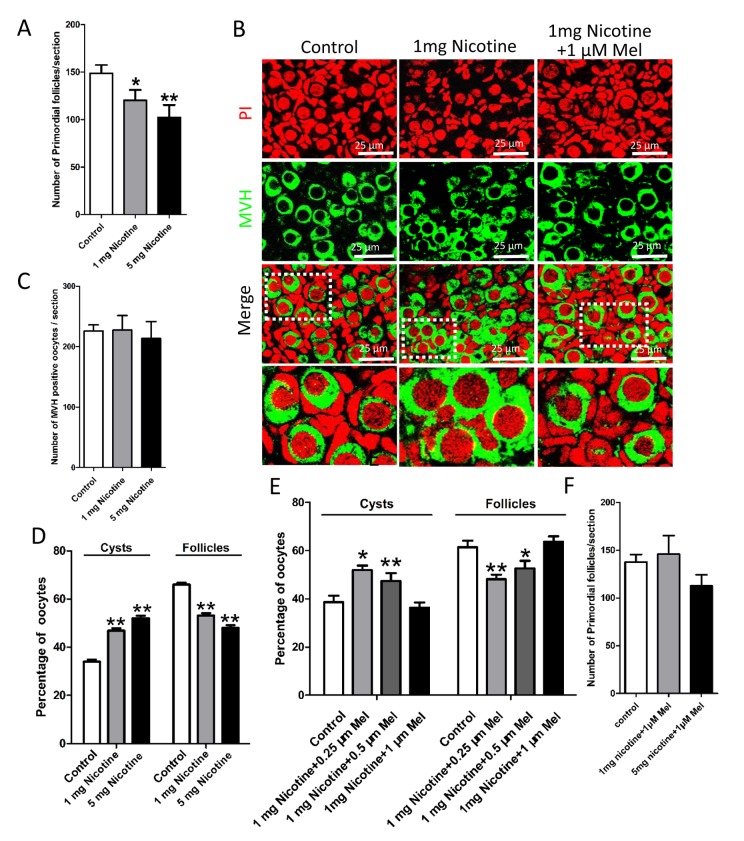
**Melatonin promotes the delay of cyst breakdown and primordial follicles assembly progression in nicotine-exposed ovaries.** (**A**) Number of primordial follicles in one section after 5 days intraperitoneally injected nicotine at incresing dosage (1 and 5 mg per kg of body weight per day). (**B**) Representative image of germ cell cyst breakdown and primordial follicle assembly alignment in control, intraperitoneally injected 1 mg/kg nicotine and intraperitoneally injected 1 mg /kg nicotine plus 1μM melatonin pups. Oocytes are stained green with anti-Mvh antibody, nuclei of oocytes and pregranulosa cells are stained red with PI. (**C**) Number of oocytes in one section after 5 days intraperitoneally injected nicotine. (**D**) Percentage of oocytes in cyst and follicle after 5 days intraperitoneally injected nicotine at incresing dosage. (**E**) Percentage of oocytes in cyst and follicle after 5 days intraperitoneally injected 1 mg /kg nicotine plus 0.25 μM, 0.5 μM and 1 μM melatonin respectively. (**F**) Number of primordial follicles in one section after 5 days intraperitoneally injected 1 mg /kg nicotine plus 1 μM melatonin and 5 mg /kg nicotine plus 1 μM melatonin respectively. The results are presented as mean±SD. All the experiments were repeated at least three times. * P < 0.05; ** P < 0.01.

**Figure 3:**
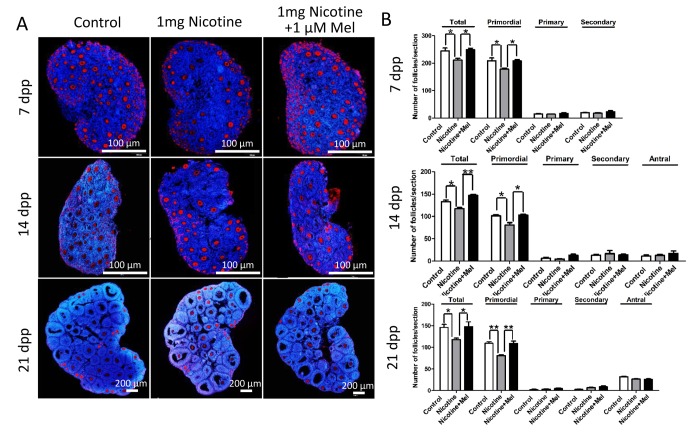
**Melatonin preserves ovarian reserve in nicotine-exposed mice.** (**A**) Representative IF for the germ cell marker Mvh in ovary tissue sections of control, intraperitoneally injected 1 mg /kg nicotine and intraperitoneally injected 1 mg /kg nicotine plus 1 μM melatonin mice after 7 dpp, 14 dpp and 21 dpp. (**B**) Quantification of the number of primordial, primary, secondary and antral follicles in control, intraperitoneally injected 1 mg /kg nicotine and intraperitoneally injected 1 mg /kg nicotine plus 1 μM melatonin mice ovaries after 7 dpp, 14 dpp and 21 dpp. All the experiments were repeated at least three times. * P < 0.05; ** P < 0.01.

Histological observations of the ovaries revealed lower numbers of the total follicle population, in particular of PFs, in the ovaries exposed to either nicotine concentrations in comparison to control (Figs. 2A and 2B); this effect was associated with a significant reduction of germ cell cyst breakdown but did not affected the total oocyte number (Fig 2C). In the control group, all large cysts broke into individual oocytes, and most of the oocytes were surrounded by granulosa cells and to form the primordial follicles. However, more germ cell cysts and fewer follicles were observed in 1 mg/kg nicotine treated group compared to control group (Fig. 2B and 2D). Follicular counts showed that 1 mg/kg nicotine treated ovaries had a significantly down-regulated percentage of oocytes in follicles (53.11 ± 2.02 vs 65.97 ± 1.59% for control group in Fig. 2D). Following the above results, we fixed 1 mg/kg bw /day nicotine for further *in vivo* studies. Surprisingly, the significantly reduction of total follicle number and PF number, between the nicotine exposed and control mice, persisted up to the age of sexual maturation around 21 dpp (Figs. 3A and 3B).

**Figure 4:**
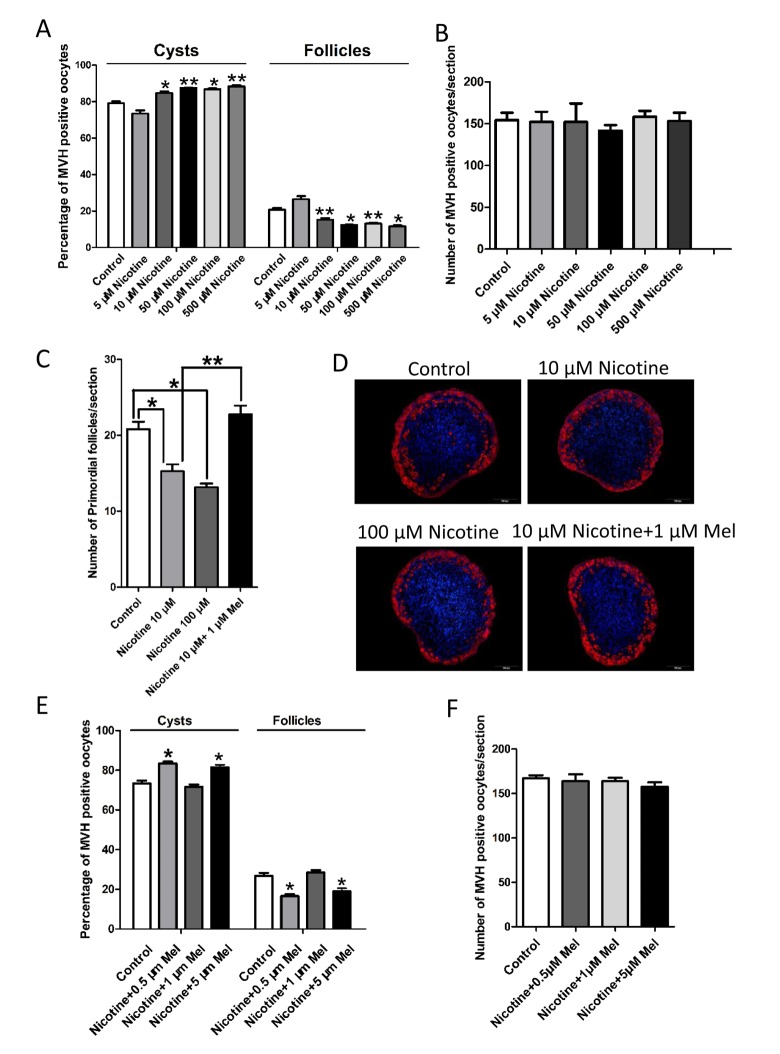
**Changes about percent of germ cell in cyst and follicle in nicotine-exposed and melatonin-treated ovaries after 5 days culture *in vitro*.** (**A**) Percentage of oocytes in cyst and follicle after the ovaries treated with nicotine at incresing dosage (5 μM-500 μM) for 5 days culture *in vitro*. (**B**) Number of oocytes in one section after the ovaries treated with nicotine at incresing dosage (5 μM-500 μM) for 5 days culture *in vitro*. (**C**) Number of primordial follicles in one section after 5 days culture *in vitro* of control, 10 μM nicotine, 100 μM nicotine and 10 μM nicotine plus 1 μM melatonin. (**D**) Representative IF for the germ cell marker Mvh in ovary tissue sections after 5 days culture *in vitro* of control, 10 μM nicotine, 100 μM nicotine and 10 μM nicotine plus 1 μM melatonin. (**E**) Percentage of oocytes in cyst and follicle after 5 days culture *in vitro* with 10 μM nicotine plus 0.5 μM, 1 μM and 5 μM melatonin respectively. (**F**) Number of oocytes in one section after 5 days culture *in vitro* with 10 μM nicotine plus 0.5 μM, 1 μM and 5 μM melatonin respectively. The results are presented as mean±SD. All the experiments were repeated at least three times. * P < 0.05; ** P < 0.01.

*In vitro* culture of 0 dpp ovaries for 5 days in the presence of 10-500 μM nicotine gave similar results (Figs. 4A-4D). Since 10 μM was the lowest nicotine concentration causing PF assembly impairment without affecting the oocyte number (Figs. 4A and 4B), we chosen this concentration for further *in vitro* studies.

### Melatonin prevents impaired folliculogenesis caused by nicotine

In this paper, we found that oocytes of primordial and primary follicles of neonatal ovaries showed from weak to moderate immune positivity for the melatonin receptor type 1 (MT1) protein. Moreover, a moderate immunostaining of MT1 in granulosa cells of primary follicles was observed (Fig. 1B).

Strikingly, we found that administration of melatonin, in the range of 0.25-1 μM, after treated with 1 mg /kg nicotine, prevented almost completely the effects of this latter on the pup’s ovaries (Figs. 2B and 2E). Expectedly, melatonin significantly increased the proportion of oocyte in follicles in nicotine-exposed ovaries to the control comparable level, especially in the 1 μM melatonin group (Fig. 2E). However, changes between different nicotine concentrate treated groups in the total oocyte number were not statistically significant (Fig. 2F). Thus 1 μM concentrate administration of melatonin was used in the subsequent *in vivo* study. In addition, melatonin preserved the follicle population at control level up to 21 dpp (Figs. 3A and 3B). In this regard, we observed that after melatonin treatment at 7 dpp, 14 dpp and 21 dpp, no significant difference about total follicle number and PF remained between the control and melatonin treatment mice, but even after 21 dpp there were also remain significantly different in total follicle number (118 ± 6 vs146 ± 14 for control group in Fig. 3B) and PF (81 ± 4 vs 109 ± 8 for control group in Fig. 3B) between the nicotine exposed and control mice (Figs. 3A and 3B). These results verified that the ovaries of pups which melatonin treatment after nicotine-exposed were able to recover a normal primordial follicle pool.

In this paper, 1 μM melatonin also prevented the 10 μM nicotine *in vitro* effect on the follicle assembly (Figs. 4E and 4F).

### Melatonin rescues the expression of oocyte specific genes and ovarian somatic cells proliferation impaired by nicotine

**Figure 5:**
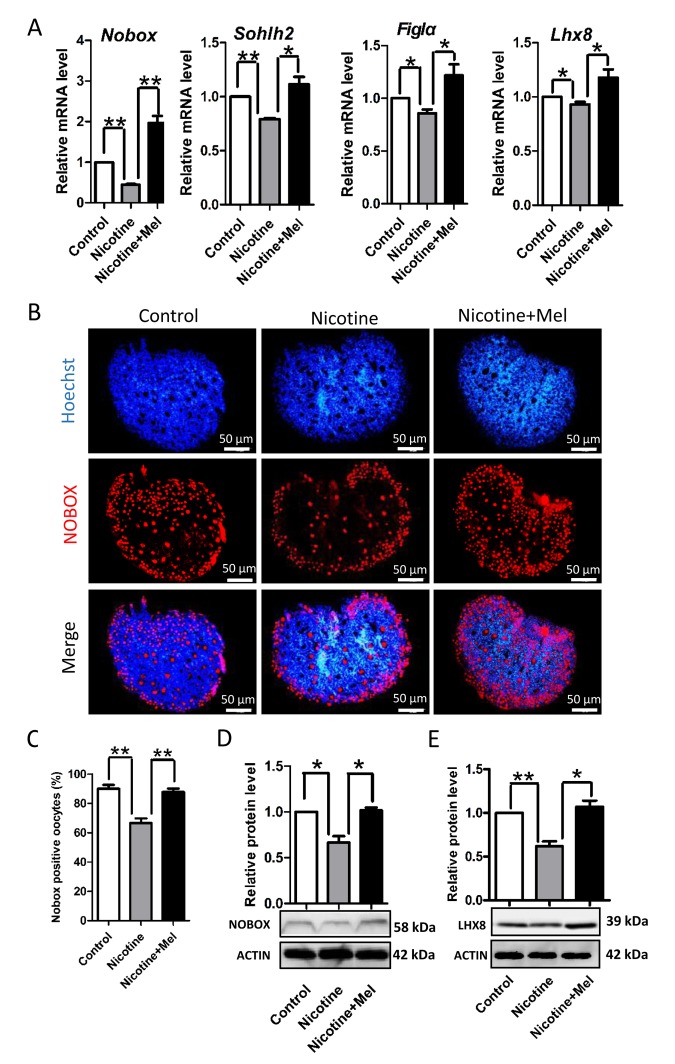
**Effect of melatonin on oocyte-specific transcription factors *Figla*, *Nobox*, *Sohlh2* and *Lhx8*, which known to be critical for the formation of primordial follicles, in nicotine-treated ovaries.** (**A**) Quantitative RT-PCR for *Nobox*, *Sohlh2*, *Figla* and *Lhx8* mRNA levels in control, intraperitoneally injected 1 mg /kg nicotine and intraperitoneally injected 1 mg /kg nicotine plus 1 μM melatonin ovaries. (**B**) Representative IF for the oocyte-specific transcription factor NOBOX in tissue sections of 5 dpp ovaries from control, intraperitoneally injected 1 mg /kg nicotine and intraperitoneally injected 1 mg /kg nicotine plus 1 μM melatonin Pups. (**C**) Percentage of oocyte showing strong NOBOX staining in ovary tissue sections. (**D**) Level of NOBOX protein in control, intraperitoneally injected 1 mg /kg nicotine and intraperitoneally injected 1 mg /kg nicotine plus 1 μM melatonin ovaries. (**E**) Level of LHX8 protein in control, intraperitoneally injected 1 mg /kg nicotine and intraperitoneally injected 1 mg /kg nicotine plus 1 μM melatonin ovaries. The results are presented as mean±SD. All the experiments were repeated at least three times. * P < 0.05; ** P < 0.01.

In order to clarify the mechanisms through which nicotine or melatonin may affect the formation of primordial follicles, In subsequent series of experiments, we found that nicotine affected the expression of oocyte genes, such as factor in the germline alpha (*Figla*) [48], newborn ovary homeobox *(Nobox*) [49], spermatogenesis and oogenesis helix-loop-helix 2 (*Sohlh2*) [50,51] and LIM homeobox 8 (*Lhx8*) [52,53], and ovarian somatic cell proliferation which are known to be crucial for PF assembly [6,46,47]. Actually, nicotine injection caused a significant decrease in the ovarian expression of oocyte specific genes such as *Figla*, *Nobox*, *Sohlh2* and *Lhx8* at mRNA levels (Fig. 5A) and for NOBOX and LHX8 also at protein level (Figs. 5B-5E). Noteworthy, melatonin injection prevented such decrease (Figs. 5A-5E).

**Figure 6:**
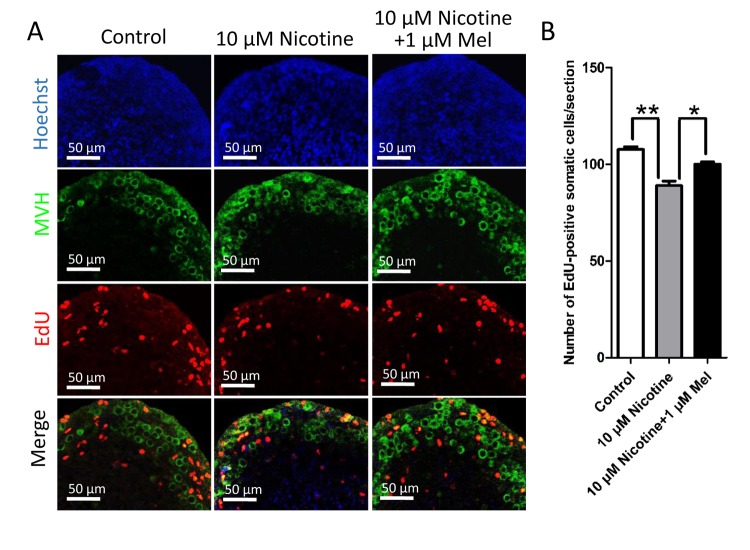
**Effect of melatonin on somatic cell proliferation during primordial folliculogenesis in nicotine-exposed ovaries cultured *in vitro* for 5 days.** (**A**) EdU histochemistry (red) in representative tissue sections of ovaries cultured *in vitro* from control, 10 μM nicotine and 10 μM nicotine plus 1 μM melatonin. (**B**) Number of EdU positive somatic cells in each section. The results are presented as mean±SD. All the experiments were repeated at least three times. * P < 0.05; ** P < 0.01.

During the first days after birth, the proliferating precursors of granulosa cells surround the oocyte cysts to form the PFs of the ovarian reserve located in the ovarian cortex [46,47]. Using *in vitro* ovary culture, we found that the continuous presence of nicotine in the medium reduced the number of proliferating ovarian somatic cells revealed by staining the incorporated EdU at fifth days of culture (Control: 107.67±2.52; nicotine: 89±4.00), also in this case, the contemporary addition of melatonin prevented the nicotine effect (99.67±2.57) (Fig. 6).

### Melatonin reduces ROS production and autophagy induced by nicotine

In a final series of experiments, we investigated the molecular mechanisms that could explain the nicotine effects on the pup ovaries focusing on the drug’s capability to induce oxidative stress and autophagy in various types of cells, including mouse granulosa cells [17,19,20,54–56].

**Figure 7:**
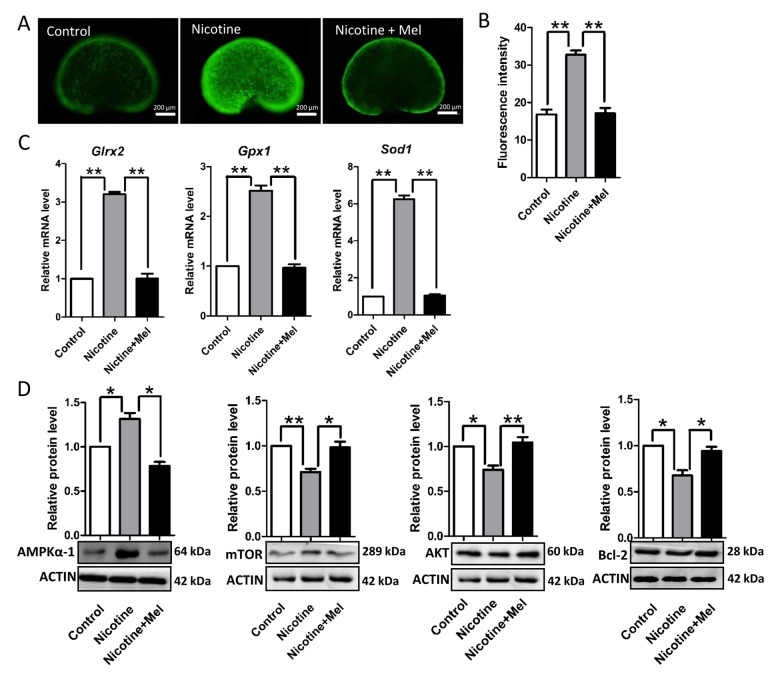
**Melatonin decrease ROS levels in nicotine-treated ovaries *in vivo*.** (**A**) Representative images of ROS levels in control, intraperitoneally injected 1 mg /kg nicotine and intraperitoneally injected 1 mg /kg nicotine plus 1 μM melatonin ovaries. (**B**) Fluorescence intensity of ROS levels. (**C**) Level of mRNA of genes involved in oxidative stress, *Glrx2*, *Gpx1* and *Sod1*, in control, intraperitoneally injected 1 mg /kg nicotine and intraperitoneally injected 1 mg /kg nicotine plus 1μM melatonin ovaries. (**D**) Protein level of AMPKα-1, mTOR, AKT and BCL-2 in control, intraperitoneally injected 1 mg /kg nicotine and intraperitoneally injected 1 mg /kg nicotine plus 1 μM melatonin ovaries were detected by western blot. The results are presented as mean±SD. All the experiments were repeated at least three times. * P < 0.05; ** P < 0.01.

**Figure 8:**
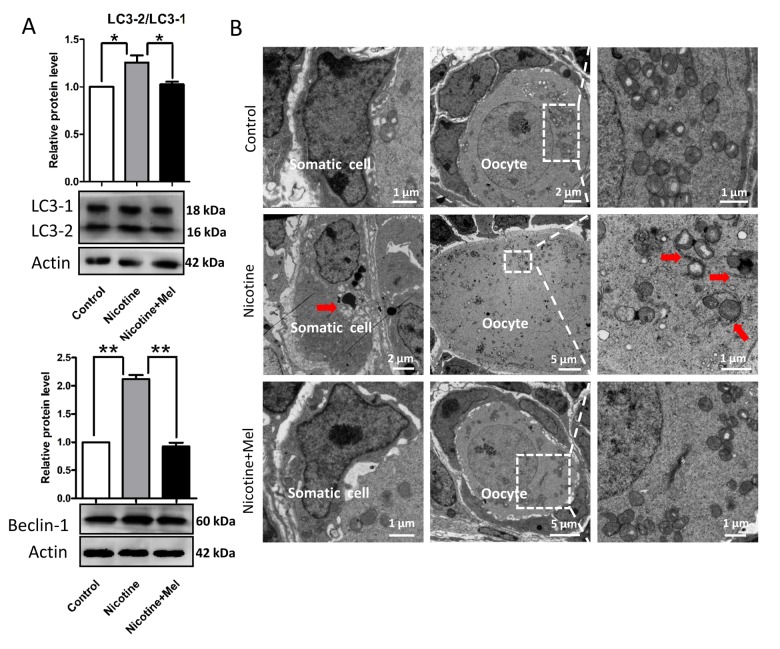
**Melatonin suppresses autophagy in nicotine-exposed ovaries.** (**A**) Protein level of LC3-2/LC3-1 and Beclin-1 in control, intraperitoneally injected 1 mg /kg nicotine and intraperitoneally injected 1 mg /kg nicotine plus 1 μM melatonin pups were detected by western blot. (**B**) Autophagosomes (arrow) in the somatic cell and oocyte cytoplasm in control, intraperitoneally injected 1 mg /kg nicotine and intraperitoneally injected 1 mg /kg nicotine plus 1 μM melatonin pups ovaries with TEM. The results are presented as mean±SD. All the experiments were repeated at least three times. * P < 0.05; ** P < 0.01.

We found that nicotine injection increased the level of ROS in the pup ovary (Figs. 7A-7B), elevating, at the same time, the transcript level of anti-oxidative enzymes such as *Girx2*, *Gpx1* and *Sod1* (Fig. 7C). Moreover, up-regulation of expression of AMP-activated protein kinase α-1 (AMPKα-1), a master regulation of the cell energy homeostasis and downregulation of AKT, mTOR and BCL-2 proteins (Fig. 7D), two events usually associated to autophagy (for a review, see Alers et al., 2012) [57], suggested activation of this process in the ovaries of the nicotine exposed pups. This was also supported by the increased ratio of the amount of the autophagosome membrane-associated light chain 3-II (LC3-II) and the cytosolic LC3-I proteins and up-regulation of BECLIN1 protein, two other autophagy markers, in the nicotine exposed ovaries (Fig. 8A). At the same time, TEM observations showed the presence of autophagosomes much more often in the cytoplasm of oocytes of ovaries of pups exposed to nicotine than unexposed and melatonin treated (Fig. 8B). Thus, the injection of nicotine plus melatonin in the pups appeared to significantly decrease both ROS increase and autophagy marker’s expression induced by the drug. On the other hand, we found that nicotine did not increased the number of apoptotic cells in the ovaries, there were rare cells found to be positive for apoptotic markers in the control group and experimental groups (The percentage of apoptotic cells/sections: control = 4.5 ± 0.9%; nicotine = 6.1 ± 1.62%; nicotine + melatonin = 4.2 ± 1.5%) (TUNEL staining Figs. S1D and S1E). Similar results could be found in previous publications about smoke-exposed ovaries [16].

## DISCUSSION

There is a plethora of evidence linking smoking to a host of adverse health outcomes including infertility by generation of free radicals [17–19]. However, the studies in the effect of nicotine on the establishment of the primordial follicle pool are very limited. The current study provides evidence that neonatal mouse ovary development is vulnerable to daily intraperitoneal administration of nicotine, which alters germ cell cyst breakdown and primordial follicle assembly. Furthermore, we also showed exposure to nicotine impaired primordial follicle formation by activation of the autophagy pathway.

In this paper, *in vivo* nicotine treated group, the concentration of nicotine was 1 mg/kg bw/day for 5 days. Petrik’s experiment showed the steady state levels of serum cotinine (the major metabolite of nicotine) of the rats, which injected with 1 mg/kg bw/day nicotine for 14 days, is 135.9 ± 7.86 ng/ml. They pointed this cotinine concentrations were within the range reported in human pregnant smokers (21.5 – 228.1 ng/ml) [23]. Furthermore, Wang et al. proved that treated mice for 6 weeks with this concentration did not affect hemodynamic parameters or metabolic indices in the mice [54]. The results of this paper showed that exposure to nicotine decreased the numbers of primordial follicle but no difference in the number of MVH positive cells between the nicotine treatment groups and control group (Fig. 2). So we conclude nicotine exposure induces oocyte and granulosa cell autophagy and ultimately depletion of the quality of oocyte and inhibit the proliferation of granulosa cells.

Our experiment are in line with earlier reports that nicotine, at concentrations relevant to those found in smokers, leads to increased levels of reactive oxygen species (ROS) [17,20,54,55]. Otherwise, this paper also revealed that nicotine exposure induced significant changes in the expression of proteins involved in: 1) induction of the autophagy pathway (AMPK); 2) inhibition of antiautophagic proteins (BCL-2, AKT and mTOR); 3) autophagy proteins BECLIN-1 and LC3. Coincide with our findings, oxidant-mediated activation of AMPKα-1 has also been shown to occur in cultured vascular smooth muscle cells exposed to nicotine [54]. In addition, there is emerging evidence demonstrating that oxidative stressors produced by cigarette smoke have been shown to increase AMPKα-1 expression [19,56]. Recent work shows that although best known for AMPK’s effects on metabolism [37], but it has many other functions, including regulation of autophagy, cell polarity, cell growth and proliferation [19,58]. Taken together, these results indicated that nicotine exposure induced oxidative stress, which trigger autophagic cascade via the AMPK together with inhibition of antiautophagic markers AKT and mTOR.

Papers performed in various mammalian species reported that melatonin protects the adult ovary from the harmful effects of a variety of treatments and compounds, including nicotine [30,32–34,36,42]. Melatonin is able to scavenge the most toxic free radical as a robust antioxidant [59]. We thus propose that melatonin could ameliorate nicotine induced ovary deterioration. As expected, in this paper melatonin treatment substantially restored the primordial follicle assembly, suggesting that melatonin indeed has the potential to improve the primordial follicle pool quality. These effects are likely mediated by the melatonin receptor MT1 reported to be present in oocytes, granulosa and theca cells of primary and antral follicles in adult mouse ovary [30] and that started to be expressed at birth [60]. In addition, other studies demonstrated that MT1 is involved in the regulation of a variety of reproductive activity [30,61,62].

Primordial follicle assembly requires interactions between oocytes and the surrounding pregranulosa cells. The proliferative somatic cells in and near the ovarian surface epithelium serve as the granulosa cell precursors, and pregranulosa cell proliferation is necessary for primordial follicle assembly and support of the enclosed oocytes [46,47]. We found fewer proliferative somatic cells in the cortex of ovaries treated with nicotine. This indicates that nicotine exposure suppresses pregranulosa cell proliferation and impairs primordial folliculogenesis. This observation may be a downstream effect of oxidative stress, because cigarette smoke treatment leading to oxidative stress and activation of autophagy in granulosa cells [17].

In conclusion, the present results show that nicotine exposure can adversely affect the establishment of the ovarian reserve in the mouse mammalian model, likely by locally inducing cellular stress that alters processes crucial for PF formation (germ cell cyst breakdown, expression of oocyte specific genes, proliferation of pre-granulosa cells) and that melatonin, however, thanks probably to its well documented antioxidant properties [30,35,42,63], is able to counteract such effects.

## MATERIALS AND METHODS

### Nicotine and melatonin administration

The CD-1 mice used for all experiments were purchased from the Institute for Drug Control (Qingdao, China) and housed in the Qingdao Agricultural University Animal Care Facility with free access to water and food under a 12 h: 12 h light - dark cycle at a constant temperature (23±2 °C) and humidity (50%). Eight-week-old female mice were mated with fertile males. Mating was timed overnight and the appearance of the vaginal plug in the next morning was considered as 0.5 days post-coitus (dpc). Birth usually occurred on 19.5 dpc and was designated as 0 day post-partum (dpp).

0 dpp pups were separated according to sex. To examine the effect of nicotine (Sigma Chemical Co., 613207, St. Louis, MO, USA), the female pups from the same litter were randomly assigned to three groups: control group, low-dose nicotine exposed group (1 mg/kg body weight (bw) /day), high-dose nicotine exposed group (5 mg/kg bw /day). Nicotine doses of 1 mg/kg bw /day and 5 mg/kg bw/day, were selected according to previous experiments [23,54]. Nicotine was dissolved in physiological saline and pH was adjusted to 7.4. Chemicals was injected into female 0 dpp pups intraperitoneally at 9:00 AM every day for 5 days (equal to 4 dpp), an equal volume of physiological saline was administrated to control pups. Melatonin (Sigma, M5250) (0.25-1 μM) was injected into pups intraperitoneally soon after nicotine injection.

All experiments were performed in accordance with institutional and national guidelines and regulations and were approved by the Ethics Committee of Qingdao Agricultural University.

### Ovary culture

0 dpp ovaries were collected and cultured as described previously [64]. Briefly, ovaries were collected and cultured on Millicell-PC membrane inserts (Millipore, PIHA01250, Medford, MA, USA) with medium filling only the lower chamber for 5 days (equal to 4 dpp); the medium was removed from the lower chamber until only a thin film covered the ovaries. The culture medium was Dulbecco’s Modified Eagle’s Medium (DMEM)/F12 (Hyclone, SH30023.01B, Beijing, China) and α-Minimum Essential Medium (α-MEM; Hyclone, SH30265.01) (1:1) supplemented with 10% fetal bovine serum (FBS; Gibco, 10099-141, USA), 50 μg/ml insulin-transferrin-selenium A mix (ITA, Gibco, 41400-045), 100 U/mL penicillin G, 100 mg/ml streptomycin sulfate, 110 μg/ml sodium pyruvate. Ovaries were treated with nicotine (5 μM - 500 μM) and nicotine plus melatonin (0.5 μM - 5 μM) as indicated. Nicotine doses were selected according to previous *in vitro* experiment performed by Sudheer et al. [55] and Kim et al. [65]. The medium was equilibrated for 2 h prior to use in a 37 °C incubator with 5% CO_2_, and changed every 48 h with replacement of half the complete medium with fresh medium. At the end of this culture period, the ovarian tissues were collected only for analyzing EdU proliferation assay in this paper.

### Immunofluorescence and TUNEL

Immunofluorescence (IF) was performed on paraffin tissue sections of the ovaries as previously described [66]. In brief, ovaries were dissected and immediately fixed in 4% paraformaldehyde overnight. The samples were processed following standard histological procedures for paraffin embedding and serially sectioned at 5 μm. The sections were heated at 60 °C in an air oven for 2 h, then immediately washed in xylene and rehydrated through a graded series of ethanol and soaked in PBS. Finally, they were transferred in 0.01 M sodium citrate buffer at high temperature (95 °C) for 10 min. After 1 h blocking with BDT (3% bovine serum albumin (BSA, Solarbio, A8020, Beijing, China), 10% normal goat serum in TBS), the sections were incubated with primary antibodies (Table S1) in a humidified atmosphere overnight at 4 °C. Cy3-labeled goat anti-rabbit (Beyotime, A0516, Nantong, China) or FITC-conjugated goat anti-mouse (Beyotime, A0568) secondary antibodies were used at dilution of 1:150 for 30 min at 37 °C in the dark. Counterstaining was performed with Hoechst33342 (Beyotime, C1022) or PI (Solarbio, P8080). Oocytes were distinguished from somatic cells for their positivity to the germ cell specific protein MVH (mouse vasa homolog) and follicles classified according to Liang’s paper [67].

Apoptosis was evaluated using the Bright Red Apoptosis Detect Kit (Vazyme, A113-02, Nanjing, China). Briefly, the paraffin sections of ovaries were heated at 60 °C for 2 h followed by washing in xylene and rehydration through a graded series of ethanol, and soaked in PBS. The sections were treated with proteinase K for 15 min at room temperature, rinsed twice with PBS and incubated with 100 μl of the TUNEL reaction mixture for 60 min at 37 °C in the dark, for negative control, only 100 μl of label solution was added. Counterstaining was performed with Hoechst33342.

After IF or TUNEL staining, 5 μm serial sections stained with antibodies were ordered sequentially on slides and double-blind counts performed every fifth sections [13]; a minimum of six different ovaries from each group were scored. Germ cells were distinguished from somatic cells based on stained positive for germ cell specific protein MVH (mouse vasa homolog). Primordial follicles were classified as an oocyte partially surrounded by either squamous granulosa cells or squamous and cuboidal granulosa cells. Primary follicles contained a small oocyte completely surrounded by a single layer of cuboidal granulosa cells. All primordial and primary follicles and germ cell cysts were counted regardless of the presence or absence of the oocyte nucleus [67].

### EdU proliferation assay

The percentage of cultured ovarian somatic cells in S-phase was evaluated by measuring EdU incorporation using the Cell - Light EdU DNA cell proliferation kit (RiboBio, C10371-1, Guangzhou, China) according to the manufacturer’s instructions and as described [16]. Briefly, EdU (50 nM) was added to the culture medium 2 h before fixation. Paraffin sections and IF were performed as described above. The slides were incubated with the staining reaction mix for 30 min. Hoechst33342 was used to visualize nuclei. Slides were also immunostained with anti-MVH antibody (Abcam, ab13840, USA). All slides were observed under a fluorescence microscope (Olympus, BX51, Tokyo, Japan) and proliferative somatic cells in the ovarian peripheral cortex scored as reported above.

### Determination of ROS level

To determine the levels of intracellular ROS production, control and chemicals treated pups were dissected and fresh ovaries were incubated with the oxidation-sensitive florescent probe dichlorodihydrofluorescein (DCFH) for 30 minutes at 37 °C in D-PBS that contained 10 μM DCFH diacetate (DCFH-DA) (Beyotime, E004). Then, they were washed three times in D-PBS containing 0.1% BSA and placed on glass slides. The measurement of the fluorescent intensity in each ovary was carried out immediately by a fluorescence microscope (Olympus, BX51).

### RT-qPCR

The extraction of total RNA from tissues was performed with RNAprep pure Micro Kit (Aidlab, RN07, Beijing, China) according to the manufacturer’s instructions and then reverse-transcribed into cDNA using TransScript One-Step gDNA Removal and cDNA Synthesis SuperMix (TransGen, AT311-03, Beijing, China). Thermal cycler program was set as 50 min at 42 °C, 65 °C for 15 min, and finally a cooling step at 4 °C. Real time quantitative PCR (RT-qPCR) was performed using a Light Cycler real-time PCR instrument (LC480; Roche, Basel, Switzerland) using Light Cycler SYBR Green I Master Mix (Roche, 04887352001) according to the manufacturer’s instructions. The primers (Table S2) used were designed with Primer Express software (Applied Biosystem) with *β-actin* used as housekeeping positive control for amplification, the reactions of which were performed in 20 μl reaction volume containing 2 μl cDNA, 10 μl of SYBR green master mix, 0.4 μl of each primer forward and reverse gene (20 μM), and 7.2 μl of nuclease-free water per sample. The PCR conditions were as follows: 10 min at 95 °C, followed by 35 cycles at 95 °C for 10 s, 60 °C for 30 s and finally a cooling step at 4 °C. Each sample had 3 technical replicates, and the reactions were performed in triplicate for each gene. Gene expression changes were analyzed by the 2^−△△Ct^ method and normalized to *β-actin*.

### Western blot

Western blot analysis was performed according to the procedure previously described [12]. Briefly, total proteins were extracted from tissues using RIPA lysis solution (Beyotime, P00113B) for 30 min on ice with frequent vortexing, then 5 μl/ovary of sodium dodecyl sulfate-PAGE (SDS-PAGE) sample loading buffer was added and the samples were boiled for 5 min. The lysates were collected by centrifugation at 12,000 rpm for 5 min at 4 °C. The proteins were separated by SDS-PAGE with a 4% stacking gel and a 10% separating gel for 50 min at 100 V and 3.5 h at 120 V, respectively, and then transferred onto polyvinylidene fluoride membrane by electrophoresis. After blocking at 4 °C overnight in TBST buffer containing 10% BSA, the membranes were incubated with specific primary antibodies for 12 h at 4 °C. Followed by washing 3 times in TBST, the membranes were incubated with horseradish peroxidase (HRP)-conjugated goat anti-rabbit or mouse IgG (Beyotime, A0208) at a dilution of 1:2000 in TBST. BeyoECL plus Kit (Beyotime, P0018) was used for exposure. *β*-ACTIN was used as control. Densitometric analyses were performed using IPWIN software.

### Transmission electron microscopy (TEM)

For TEM observations, fresh ovaries collected from the control and chemical treated mice, were immediately fixed in 2.5% glutaraldehyde in 0.2 M PBS (pH=7.2) overnight at 4 °C. The samples were processed and included in epoxypropane resin following standard TEM procedures. Serial sections were cut at 50 nm using the EM UC7 ultramicrotome (Leica, Germany), stained with lead citrate and uranium and observed under HT7700 transmission electron microscope.

### Statistical analyses

For each set of results, independent experiments were repeated at least 3 times, with data representing the mean + SEM. Data were analyzed by unpaired Student’s t-Test with 2-tailed distribution by using GraphPad Prism5 software; P < 0.05 denoted statistically significant difference, while P < 0.01 denoted highly significant difference.

## Supplementary Material

Supplementary File
