# Supplementary material for: Protective effects of melatonin against nicotine-induced disorder of mouse early folliculogenesis
Source: Aging (Albany NY). 2018 Mar 28;10(3):463–80. doi: 10.18632/aging.101405 (PMC5892698; doi:10.18632/aging.101405)
Supplement: Supplementary File [file aging-10-101405-s001.pdf]

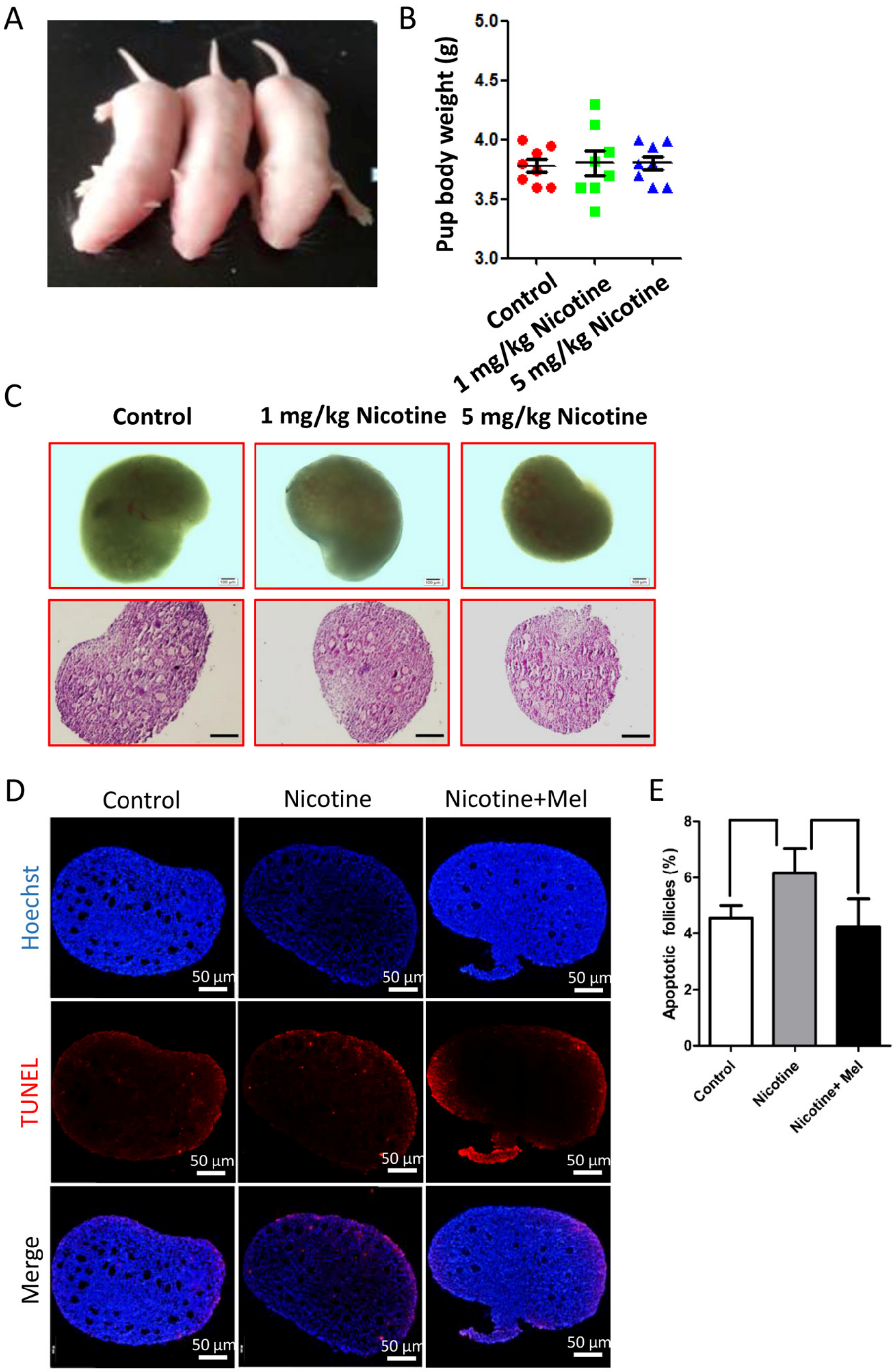

Figure S1. Nicotine-exposed pups compared with normal littermate after deal with 5 days. (**A**) Photograph of nicotine-exposed pups compared with normal littermate. (**B**) Body weight of nicotine-exposed and control pups. (**C**) Gross morphologies and representative H&E histological sections of the ovaries dissected from 4 dpp pups of the nicotine-exposed groups. (**D**) TUNEL histochemistry (red) in representative tissue sections of ovaries from normal, intraperitoneally injected 1 mg /kg nicotine and intraperitoneally injected 1 mg /kg nicotine plus 1μM melatonin pups. (**E**) The percentage of follicles with two or more TUNEL-positive cells in intraperitoneally injected 1 mg /kg nicotine and intraperitoneally injected 1 mg /kg nicotine plus 1 μM melatonin pups compared with normal pups. For each group, five ovaires were collected and follicles (n = 900) for statistical results, The results are presented as mean±SD. All the experiments were repeated at least three times. * P < 0.05; ** P < 0.01.

Table S1. Primers used for quantitative RT-PCR.

Genes	Genbanks	Forward primer sequences	Reverse primer sequences	Product Length (bp)
β-actin	NM_007393.5	TCGTGGGCCGCCCTAGGCAC	TGGCCTTAGGGTTCAGGGGGG	243
Figla	NM_012013.1	ACAGAGCAGGAAGCCCAGTA	TGGGTAGCATTTCCCAAGAG	225
Nobox	NM_130869	CTATCCTGACAGTGACAAACGCC	CACCCTCTCAGCACCCTCATTAT	251
Lhx8	NC_000069.5	CAGTTCGCTCAGGACAACAA	CCTGCAGTTCTGAAACCACA	105
Sod1	NM_001005735.1	GGGGAAGCATTAAAGGACTGA	CCACCGTGTTTTCTGGATAGA	124
Glrx2	XM_00652985	ATCGTCGTTTTGGGGGAAGT	GGAACAGTAAGAGCAGGATGTTT	152
Sohlh2	NM_028937.3	TCTCAGCCACATCACAGAGG	GGGGACGCGAGTCTTATACA	199
Gpx1	NM_000581.2	AGTCGGTGTATGCCTTCTCG	AGCTCGTTCATCTGGGTGTAGT	145
α4 nAChR	NM_015730	CTCAGATGTGGTCCTTGTC	GAGTTCAGATGGGATGCG	178
α5 nAChR	NM_176844	CATCGTTTTGTTTGATAATGC	TGCGTCCAAGTGACAGTG	90
α7 nAChR	NM_007390	GGTCATTTGCCCACTCTG	GACAGCCTATCGGGTGAG	130
α9 nAChR	NM_001081104	ACAAGGCCACCAACTCCA	ACCAACCCACTCCTCCTCTT	152
α10nAChR	NM_001081424	TCTGACCTCACAACCCACAA	TCCTGTCTCAGCCTCCATGT	168
β4 nAChR	NM_148944	CTACAGGAAGCATTAGAGG	CAGAATACACACAATCACG	146

Table S2. Antibodies used in this paper.

Antibodies	Vendor	Dilution
BCLIN-1 (WB)	Cell signaling (#3738)	1:1000
LC3B (WB)	Abcam (ab51520)	1:3000
BCL-2 (WB)	Beyotime (ab116)	1:1000
mTOR (WB)	Bioss (bs-1992R)	1:200
AMPKα-1 (WB)	Bioss(bs-1115R)	1:500
MVH (IF/WB)	Abcam(ab13840)	1:100
AKT (WB)	Cell signaling (#2920)	1:1000
NOBOX (IF/WB)	Abcam (ab41521)	1:150/1:1000
LHX8 (WB)	Abcam (ab126981)	1:1000
β-ACTIN (WB)	Abcam (ab3280)	1:1000
MT1	Novusbio(Nbp1-71113)	1:500
